# Comparison of discover cervical disc arthroplasty and anterior cervical discectomy and fusion for the treatment of cervical degenerative disc diseases: A meta-analysis of prospective, randomized controlled trials

**DOI:** 10.3389/fsurg.2023.1124423

**Published:** 2023-02-21

**Authors:** Ziqi Wang, Gan Luo, Hongwei Yu, Hui Zhao, Tianhao Li, Houzhi Yang, Tianwei Sun

**Affiliations:** ^1^ School of Medicine, Nankai University, Tianjin, China; ^2^ Graduate School of Tianjin Medical University, Tianjin, China; ^3^ Department of Spine Surgery, Tianjin Union Medical Center, Tianjin, China

**Keywords:** anterior cervical discectomy and fusion, discover cervical disc arthroplasty, meta-analysis, randomized controlled trial, degenerative disc diseases

## Abstract

**Objective:**

This study aims to evaluate the clinical efficacy and safety between Discover cervical disc arthroplasty (DCDA) and anterior cervical discectomy and fusion (ACDF) in Cervical degenerative disc diseases.

**Methods:**

Two researchers independently conducted a search of PubMed, EMBASE, and Cochrane Central Register of Controlled Trails (CENTRAL) for randomized controlled trials (RCTs) following the Cochrane methodology guidelines. A fixed-effects or random-effects model was applied based on different heterogeneity. Review Manager (Version 5.4.1) software was used to perform data analysis.

**Results:**

A total of 8 RCT studies were included in this meta-analysis. The results indicate that the DCDA group had a higher incidence of reoperation (*P* = 0.03) and a lower incidence of ASD (*P* = 0.04) than the CDA group. There was no significant difference between two groups regarding NDI score (*P* = 0.36), VAS ARM score (*P* = 0.73), VAS NECK score (*P* = 0.63), EQ-5D score (*P* = 0.61) and dysphagia incidence (0.18).

**Conclusion:**

DCDA and ACDF have similar results in terms of NDI scores, VAS scores, EQ-5D scores, and dysphagia. In addition, DCDA can reduce the risk of ASD but increases the risk of reoperation.

## 1. Introduction

Spinal degeneration is very common in the population. Some studies have shown that at age 50, about 80%–90% of people will show disk degeneration on magnetic resonance imaging (MRI) (1, 2). Degeneration of the cervical spine may further affect the cervical nerve roots and the cervical spinal cord, ultimately causing clinical symptoms.(3) The number of patients requiring surgical treatment for symptomatic cervical degenerative disease has increased in recent years. In the study by Kazuyoshi et al. (4), the number of patients who eventually required surgery for CDDD increased 1.9 times over 12 years. Previous research on this type of disease is extensive, and its treatment is varied.(5–7) Among them, ACDF is a recognized and effective means for treating this disease (8, 9). The success rate of ACDF and its clinical efficacy have been well demonstrated (10–12). However, ACDF can also cause some adverse effects. The complications that have been previously reported include postoperative development of angulation deformity, dysphagia, bone graft or instrumentation extrusion, adjacent segment degeneration, and postoperative mechanical instability of the cervical spine (13–15). In addition, biomechanical studies have also shown an increased incidence of disc degeneration at the level adjacent to the spinal fusion segment (16, 17). Hilibrand et al. (18) reported that approximately 25% of patients would develop further degeneration of the adjacent segment within 10 years after ACDF surgery. The emergence of CDA is to address some of the complications caused by ACDF. The theoretical advantage of cervical arthroplasty is its ability to preserve more segmental mobility, reducing the incidence of adjacent segmental degeneration and avoiding some of the limitations of ACDF (19, 20). However, CDA is not a perfect substitute for ACDF, as heterotopic ossification and implant migration or subsidence are common complications of CDA (21, 22). Multiple studies have compared the clinical efficacy and adverse events of CDA and ACDF, but the conclusions drawn from these studies are inconsistent (23, 24). Many meta-analyses integrate data from related articles in order to obtain more accurate results. Still, the type of prosthesis is often not taken into account in these articles (25–27). However, a meta-analysis by MD et al. (28) revealed that the clinical efficacy and the incidence of adjacent segmental disorders differed between the different classes of prostheses. Therefore, this article is dedicated to comparing one of the prostheses (Discover disc prosthesis) with ACDF.

## 2. Materials and methods

### 2.1. Search strategy

We performed a systematic review and meta-analysis according to the Preferred Reporting Items for Systematic Reviews and Meta-Analyses (PRISMA) guidelines. To make an exhaustive search of all relevant literature, we conducted a search of PubMed, EMBASE, and Cochrane Central Register of Controlled Trails (CENTRAL) for randomized controlled trials (RCTs) following the Cochrane methodology guidelines. We located studies with the following search terms: cervical disc arthroplasty, cervical disc replacement, CDA, CDR, artificial disc replacement, anterior cervical discectomy and fusion, ACDF, Discover. We did not restrict the language of the articles, and the literature search results were last updated on August 1, 2022. We also searched the reference lists of the included literature in case any relevant articles were missed. We obtained the full text of all potential articles. Two independent reviewers screened the titles and abstracts of these articles and finally determined the literature that met the criteria for this meta-analysis.

### 2.2. Criteria for selected trials

Articles that met the following criteria were included in our study: (1) An RCT comparing ACDF with Discover artificial disc replacement for cervical degenerative diseases. (2) The article contains information on at least one of the outcome indicators such as NDI score, VAS score, EQ-5D score, reoperation rate, dysphagia incidence, and ASD incidence. (3) The age of the individuals involved in the article should be older than 18 years; (4) The follow-up period in the literature should be at least >24 months. The exclusion criteria for the literature were as follows: (1) the articles were observational studies, reviews, or case reports. (2) Articles with incomplete data or continuous variables for which standard deviations were not available. (3) Repeated publication of the same data.

### 2.3. Data extraction

Two independent reviewers extracted data from the included literature. When we encountered discrepancies, we would reach a consensus by discussion. If necessary, we will combine the opinions of a third reference to make a final decision. The essential information extracted from the inclusive article includes the study design, sample size, sex distribution, age, experimental and control interventions, duration of follow-up, and outcomes. In this literature, the outcome indicators we analyzed included the NDI score, EQ-5D score, VAS score, and the number of patients presenting with dysphagia, reoperation, and ASD.

### 2.4. Quality of evidence assessment

Two researchers applying the Cochrane Collaboration tool evaluated the included literature. The content of the device consists of the following aspects: (1) random sequence generation; (2) allocation concealment; (3) blinding of participants and personnel; (4) blinding of outcome assessment; (5) incomplete outcome data; (6) selective reporting; (7) other bias. Each item is judged by high risk, low risk, or unclear risk. Disagreements will be resolved by discussion or by consulting a third author.

### 2.5. Statistical analysis

RevMan 5.4.1 (Cochrane, London, UK) was used to perform a combined analysis of the extracted data. The standardized mean difference (SMD) and its 95% confidence interval (CI) were calculated for the continuous data, and the risk ratio (RR) and its 95% CI were calculated for the dichotomous data. The heterogeneity of studies was estimated using the *I*^2^ tests: low heterogeneity (*I*^2^ < 25%), moderate heterogeneity (25% < *I*^2 ^< 50%), and substantial heterogeneity (*I*^2^ > 50%). Due to the possible difference in disease severity of patients before surgery, all patients adopt random effect model. The possibility of publishing bias was not researched because of the limited number of included studies. We defined the follow-up period of fewer than 3 years as short-term follow-up and the follow-up period of more than 5 years as long-term follow-up. The statistically significant level was set at *P* < 0.05.

## 3. Results

### 3.1. Search results

**Figure 1:**
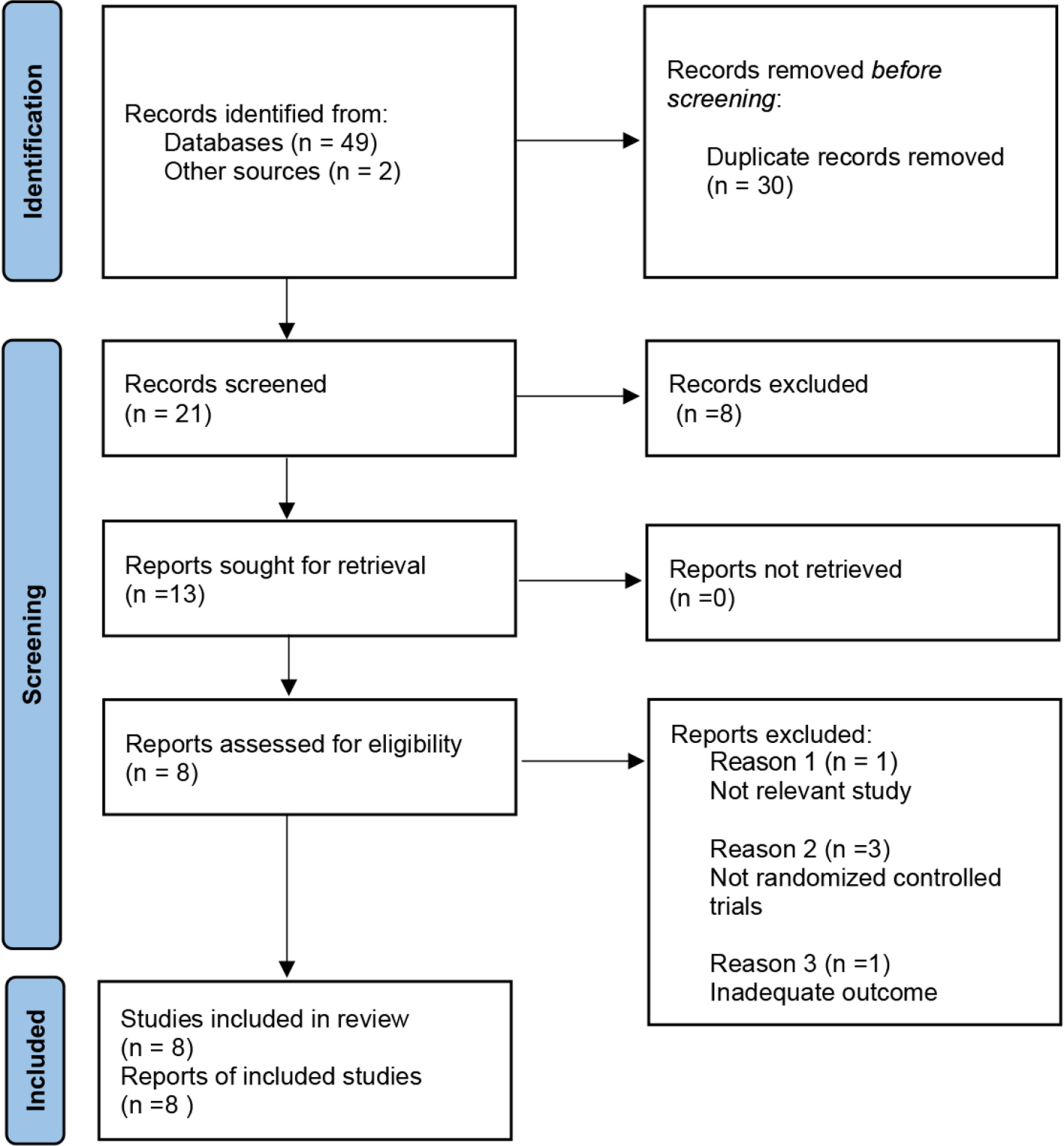
Flow diagram of the study selection process for the meta-analysis.

A total of 51 articles from PubMed, Embase, Cochrane Library, and reference lists were initially identified. 17 papers were found in PubMed, 11 were from Embase, 21 were found in Cochrane library, and 2 were from the reference lists. 30 articles were excluded because they were duplicates. By screening the titles and abstracts, 8 articles were excluded because they did not meet our indicated inclusion criteria for the literature. 5 of the remaining articles were excluded by analysis of the full text. Ultimately, 8 pieces were enrolled in this meta-analysis for qualitative and quantitative analyses (Figure 1).

### 3.2. Study characteristics

8 RCT studies (29–36) published between 2010 and 2021 were included in the present study. Except for the two data sets that differed in follow-up time, a total of 475 patients were involved in the 8 studies. The DCDA group was 227 patients, and the ACDF group was 248. Four of the included papers had a 2-year follow-up, two articles had a 5-year follow-up, and the remaining two articles had a mean follow-up of 32.4 and 38 months respectively. The characteristics of included studies are presented in Table 1.

**Table 1:** Characteristics of included studies.

Study	Patients (n)			Mean age		Sex (M/F)		Follow up	Region
	total	DCDA	ACDF	DCDA	ACDF	DCDA	ACDF		
Skeppholm 2015	151	81	70	46.7 (6.7)	47.0 (6.9)	40/41	33/37	24 months	Sweden
MacDowall 2019	153	83	70	46.9 (6.8)	47.0 (6.9)	42/41	33/37	60 months	Sweden
Qizhi 2016	30	14	16	46.79 (5.15)	48.13 (5.98)	9/5	11/6	32.4 months	China
Rožanković 2017	101	51	50	41.32 (8.8)	41.94 (9.36)	25/26	25/25	24 months	Croatia
Sundseth 2017	136	68	68	44.7 (7.2)	43.4 (6.8)	38/30	36/32	24 months	Norway
Johansen 2021	136	68	68	44.7 (7.2)	43.4 (6.8)	38/30	36/32	120 months	Norway
Coric 2010	23	16	7	—	—	—	—	38 months	America
Chen 2013	32	16	16	43.2 (10.2)	46.5 (7.9)	9/7	8/8	24 months	China

### 3.3. Risk of bias in included studies

**Figure 2:**
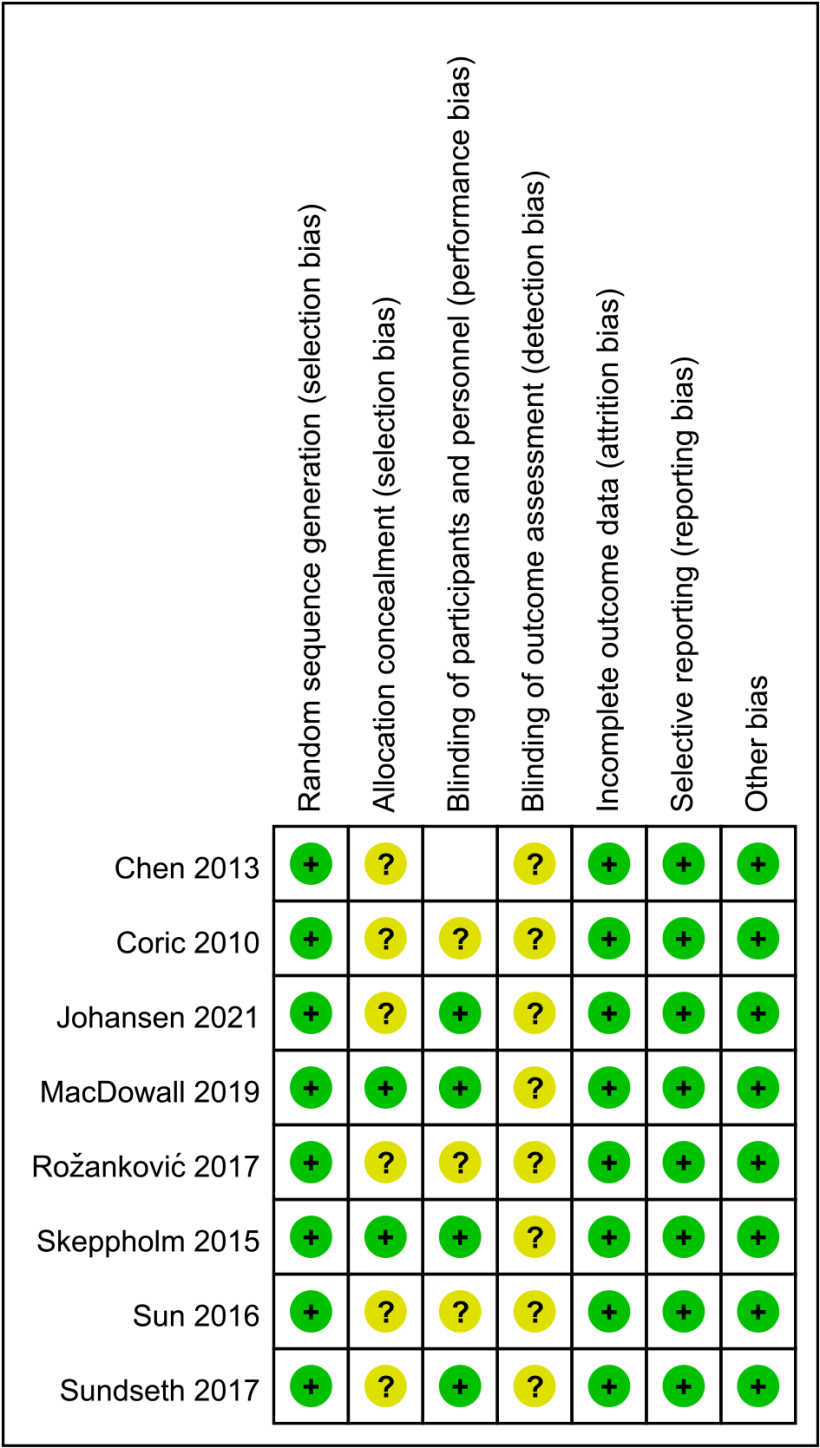
The risk of bias in the included studies was evaluated in our meta-analysis.

Among the literature included in this study, eight articles had a low risk of bias for random sequence generation. 2 of the studies described allocation concealment. The remaining six articles do not provide a detailed description of whether allocation concealment was performed. Four studies described the blinding of participants and personnel. None of the included studies described blinding to outcome assessment (Figure 2).

### 3.4. Clinical indices

#### 3.4.1. Neck disability index

**Figure 3:**
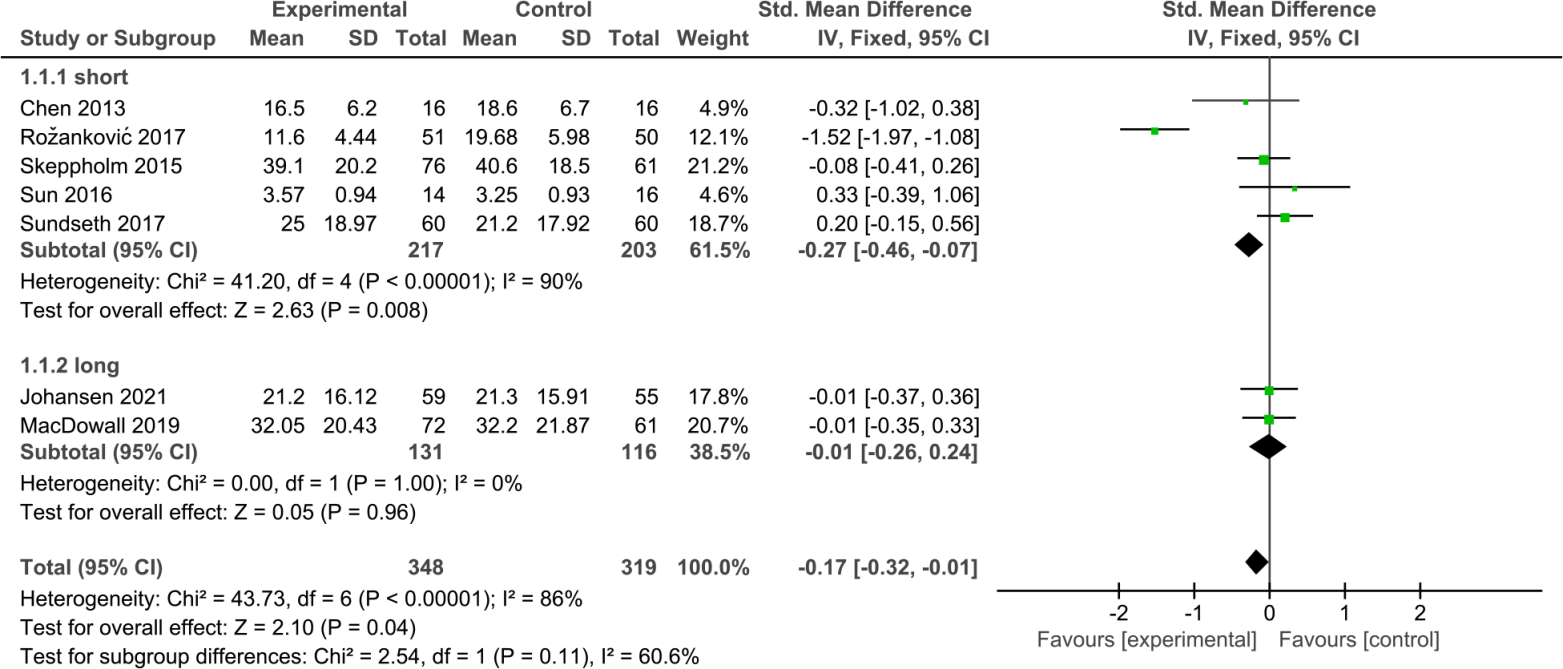
Comparison of NDI score between the discover cervical disc arthroplasty (DCDA, “experimental”) group and the anterior cervical discectomy and fusion (ACDF, “control”) group.

A total of 7 articles compared the NDI scores of the DCDA and ACDF groups. Five of these articles (29, 31, 32, 35, 36) had a 2-year follow-up period, and the other two (30, 33) had a 5-year follow-up period. In comparing short-term outcomes, the NDI score was lower in the experimental group than in the control group, but the difference was not statistically significant. (SMD, −0.29; 95% CI: −0.96–0.38, *P* = 0.40). In comparing long-term outcomes, DCDA and ACDF also did not show statistically significant differences. (SMD, −0.01; 95% CI: −0.26–0.24, *P* = 0.96) (Figure 3).

#### 3.4.2. VAS NECK

**Figure 4:**
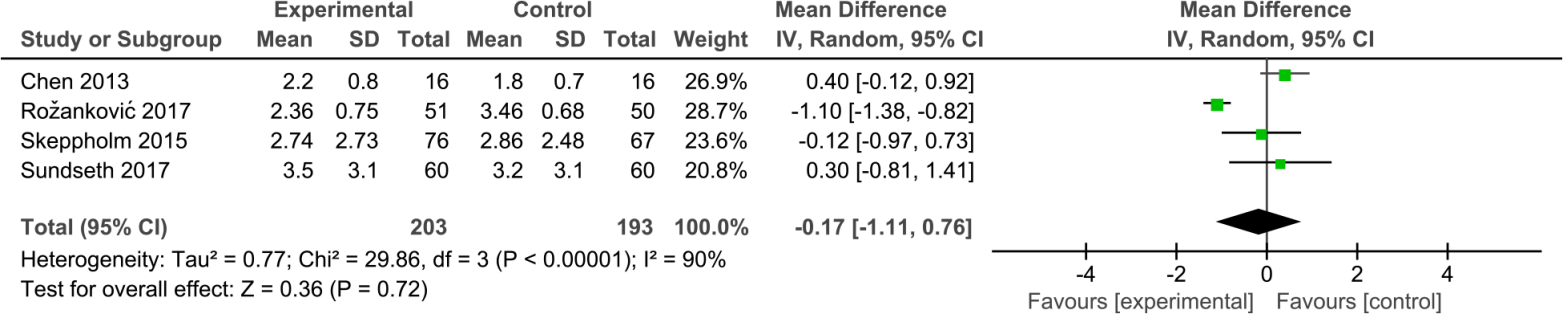
Comparison of VAS NECK score between the discover cervical disc arthroplasty (DCDA, “experimental”) group and the anterior cervical discectomy and fusion (ACDF, “control”) group.

Four studies (31, 32, 35, 36) reported mean neck pain VAS scores within the DCDA and ACDF groups, respectively. There was significant heterogeneity between the two groups (*P* < 0.00001, *I*^2^ = 89%). The results showed no statistically significant difference in neck pain VAS score between the two groups. (SMD, −0.26; 95% CI: −1.04–0.53, *P* = 0.53) (Figure 4).

#### 3.4.3. VAS ARM

**Figure 5:**
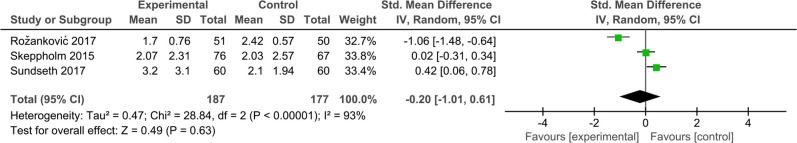
Comparison of VAS ARM score between the discover cervical disc arthroplasty (DCDA, “experimental”) group and the anterior cervical discectomy and fusion (ACDF, “control”) group.

Three studies (31, 35, 36) contributed to the analysis of the arm pain VAS score. There was significant heterogeneity between the two groups (*P* < 0.00001, *I*^2^ = 93%). No significant difference was found when comparing the arm pain scores between the two groups (SMD, −0.20; 95% CI: −1.01–1.61, *P* = 0.63) (Figure 5).

#### 3.4.4. EQ-5D

**Figure 6:**
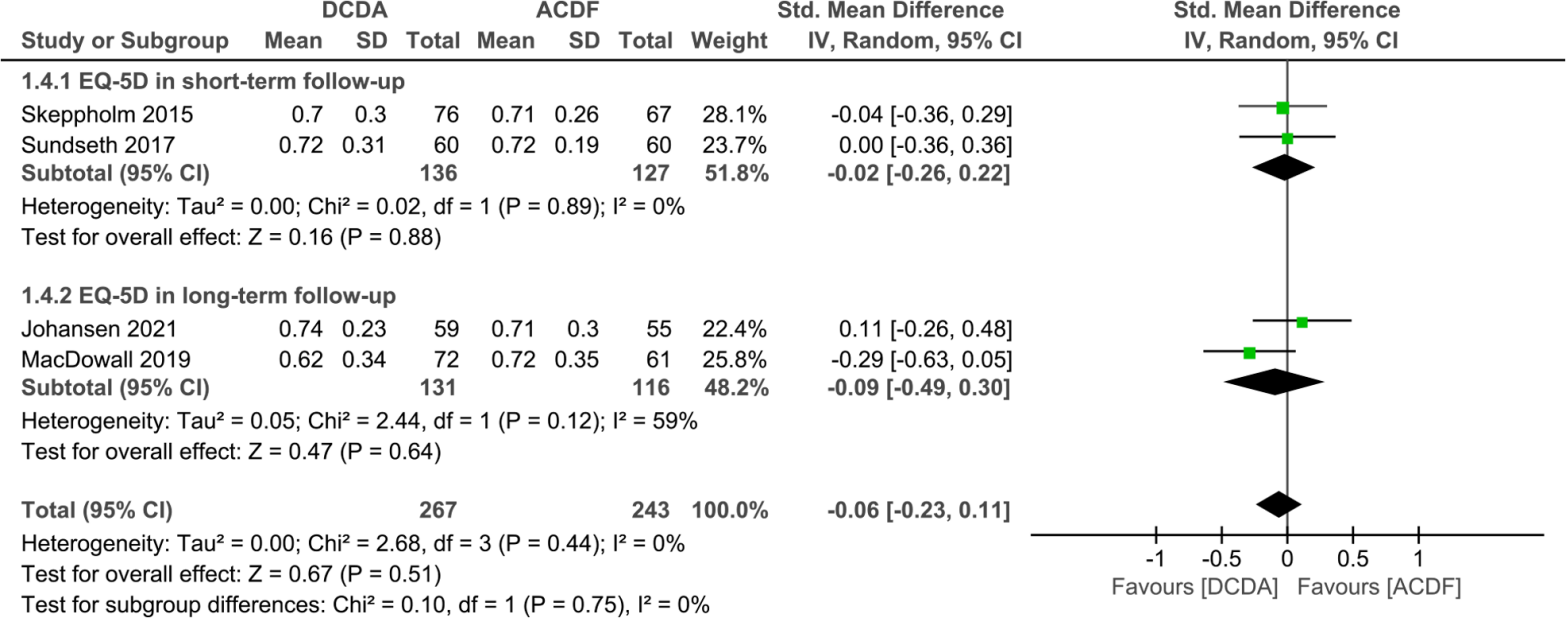
Comparison of EQ-5D score between the discover cervical disc arthroplasty (DCDA, “experimental”) group and the anterior cervical discectomy and fusion (ACDF, “control”) group.

EQ-5D score was reported in four studies (30, 33, 35, 36). Two of these articles compare short-term results, while the other two compare long-term results. The pooled results indicated that the DCDA and ACDF groups were not showing statistical differences in either long-term or short-term outcomes. (short-term, SMD, −0.02; 95% CI: −0.26–0.22, *P* = 0.88 long-term SMD, −0.09; 95% CI: −0.49–0.30, *P* = 0.64) (Figure 6).

### 3.5. Adverse events

#### 3.5.1. Dysphagia

**Figure 7:**
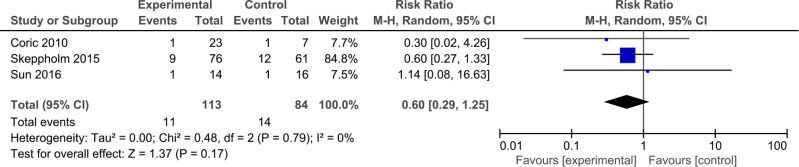
Comparison of dysphagia between the discover cervical disc arthroplasty (DCDA, “experimental”) group and the anterior cervical discectomy and fusion (ACDF, “control”) group.

Three studies with 113 patients were included to assess the incidence of dysphagia. All patients had only short-term follow-up results. There were no significant difference in short-term follow-up between DCDA and ACDF (RR: 0.60, 95% CI: 0.29–1.25, *P* = 0.17). The results were consistent across the studies (*I*^2 ^= 0%, *P* = 0.79) (Figure 7).

#### 3.5.2. Reoperation

**Figure 8:**
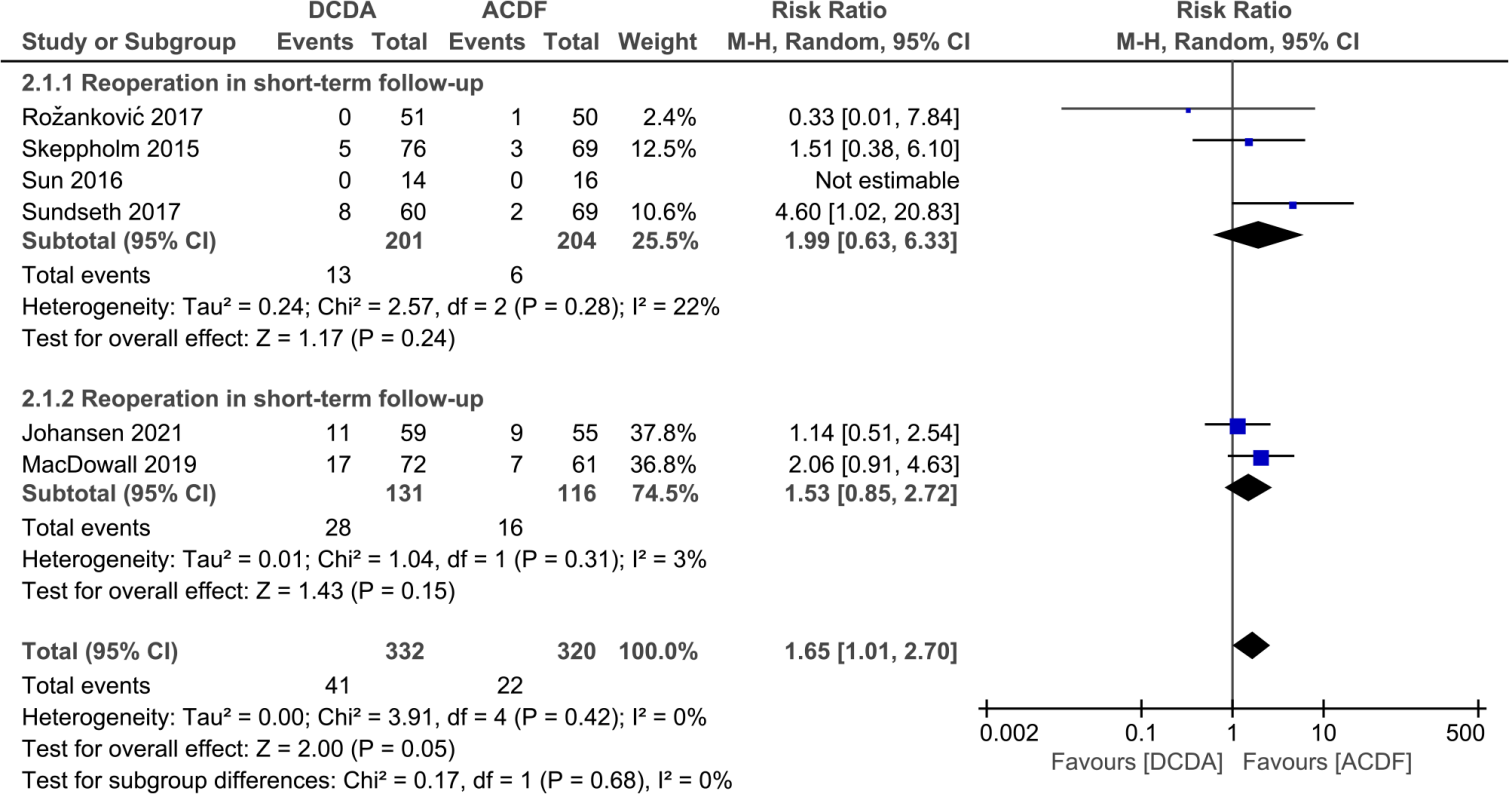
Comparison of reoperation between the discover cervical disc arthroplasty (DCDA, “experimental”) group and the anterior cervical discectomy and fusion (ACDF, “control”) group.

Six studies reported reoperations at the final follow-up. There was no significant heterogeneity between the two groups (short-term *P* = 0.28; *I*^2^ = 22%; long-term *P* = 0.31; *I*^2^ = 3%). The results showed that the reoperation rate of the DCDA group was higher than the ACDF groups, with no statistical difference in short-term and long-term outcomes. In the comparison of total outcomes, the probability of secondary surgery was higher in DCDA, and this value was statistically significant. (total, RR = 1.65, 95% CI: 1.01–2.70, *P* = 0.05; short-term, RR = 1.99, 95% CI: 0.63–6.33, *P* = 0.24; long-term, RR = 1.53, 95% CI: 1.01–2.70, *P* = 0.43) (Figure 8).

#### 3.5.3. ASD

**Figure 9:**
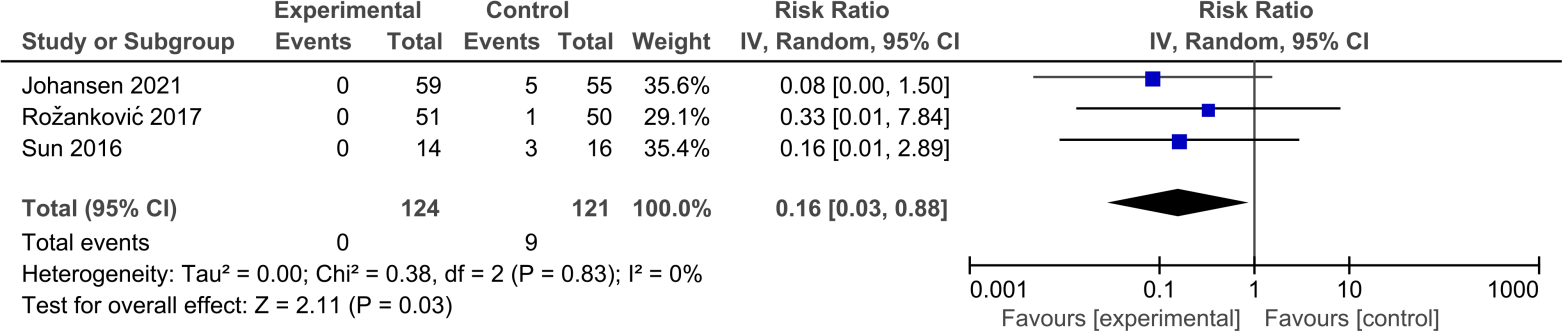
Comparison of ASD between the discover cervical disc arthroplasty (DCDA, “experimental”) group and the anterior cervical discectomy and fusion (ACDF, “control”) group.

Four articles compared the incidence of ASD, and the follow-up period for these articles ranged from 24 to 120 months. Ultimately, the results revealed that the ASD rate of the DCDA group was lower than that of the ACDF group. (RR: 0.16, 95% CI: 0.03–0.88, *P* = 0.03) (Figure 9).

### 3.6. Sensitivity analysis

Sensitivity analysis was conducted to determine whether the general trend would be significantly affected by deleting each study. Except for NDI and VAS ARM scores, no altered results were observed after each study was eliminated.

## 4. Discussion

Many meta-analyses have previously been performed comparing ACDF and CDA. Some studies (37–39) found that CDA was superior to ACDF in the comparison of NDI and EQ-5D scores and had better clinical efficacy. In addition, some other studies (40, 41) noted that the CDA and ACDF groups did not show a statistically significant difference in the incidence of ASD. However, all these studies regard CDA as a whole when comparing CDA and ACDF. In fact, CDA is a relatively broad concept, and the prostheses utilized in different studies are variable. Based on the article by Phillips et al. (42), artificial cervical discs can be classified by material into metal-on-metal, metal-on-polymer, ceramic-on-metal and ceramic-on-ceramic types. If classified according to biomechanics, the cervical prosthesis can also be further divided into three types — non-constrained,semi-constrained and constrained. Different types of prostheses have their own advantages, such as metal-to-metal types that produce less debris and metal-to-polymer prostheses that are better at simulating disc physiology and MR imaging. Coban et al. (28) have reported the differences in ASD between metal-on-metal and metal-on-polymer. In summary, comparing different prostheses with ACDF or between the various types of prostheses is reasonable. The clinical application of the Discover cervical artificial disc was first reported by Greiner–Perth et al. (43) in 2009. DISCOVER prosthesis is both a metal-on-polymer type and a non-constrained type of prosthesis, which may have advantages in terms of the incidence of heterotopic ossification and ASD. That is why we conducted a meta-analysis of eight RCTs to determine whether DCDA was superior to ACDF. The results suggest ACDF was superior to DCDA with a significantly lower incidence of secondary surgical procedures. However, the DCDA group had significantly better results in terms of the occurrence of ASD compared with the ACDF group.

The NDI is a very common and essential index used to evaluate the clinical efficacy of cervical spine surgery. Previous studies (38, 44) have found that CDA has a lower NDI score than ACDF. Meanwhile, other studies (29, 45) have also found no significant difference between DCDA and ACDF in the comparison of postoperative NDI. When applying the random effects model, the NDI between the two groups in this study did not show significant differences in both long- and short-term outcomes. In addition, the results showed a significant difference after removing the article by Rožanković et al. (31) The patients included in this article have (NDI) score of ≥30%, which was not described in the other enrolled articles. This may be the reason for the unstable results and the high heterogeneity of the index. And in the comparison of long-term outcomes, only two articles (30, 33) provided data for the 5-year follow-up period, so more high-quality long-term follow-up data need to be further investigated.

In previous articles (46, 47), the CDA and ACDF groups had similar effects in relieving arm and neck pain. Such results are also consistent with this meta-analysis. Kan et al. (48) indicated that the CDA group had lower VAS ARM and VAS NECK scores than the ACDF group. However, such differences may not have clinical significance, and patients may not be able to perceive subtle differences in scores. The results presented in this article are not robust due to the high heterogeneity. VAS focuses on the degree of pain, so patients with cervical spondylotic radiculopathy may have greater preoperative and postoperative differences. In addition, although the VAS scoring methods are all based on visual assessment, there are differences in the actual scoring details. And this difference is often not described in the articles. In the article by Skeppholm et al. (35), the VAS score is larger than the score of the standard scoring method. Therefore, we adjusted the VAS score of this article according to the standard score. All of the above reasons will have an impact on the current results. Hence, with the initiation of additional high-quality studies with longer follow-up, the VAS scores should be studied more rigorously. In the EQ-5D scores, the two groups did not show significant differences. The EQ-5D is a standardized set of scales that measure health status. The scale assesses a person's state of health through three levels on five dimensions. However, because of the small number of levels delineated, the scale is less sensitive to changes in small and medium health levels. Therefore, the application of the more accurate EQ-5D-5l scale in subsequent studies may be able to demonstrate more accurately whether there are differences between the two groups.

In the comparison of adverse events, Yao et al. (49) and Jiang et al. (50) found no significant difference in dysphagia incidence between the two groups. However, the study by McAfee et al. (51) showed that CDA was superior to ACDF in terms of dysphagia because CDA does not require strict retraction of the esophagus past the midline during the surgery procedure. Moreover, the occurrence of dysphagia is also influenced by other factors. Topical or intravenous steroid administration may also impact dysphagia rates (52). Hence, research on this issue still needs to limit more confounding factors to more accurately explore the advantages and disadvantages of DCDA and ACDF. Besides, the DCDA group showed more secondary procedures in this study. Such a result is not consistent with some previous studies. Many studies (53–55) have shown that the secondary surgical procedure for CDA is less than ACDF. Degeneration of the adjacent segment, implant events and persisting radiculopathy can all lead to reoperation.(53) Further refinement of the cause analysis may be more helpful in understanding the actual situation. In addition, this study found that the rate of ASD in the DCDA group was lower than that in the ACDF group. There are many relevant studies supporting such results. For example, a biomechanical study by Eck et al. (17) found that CDA may have less impact on adjacent segments. This biomechanical variation is why CDA is less likely to cause ASD. Reducing the effects on adjacent segments is also one of the reasons why the CDA technique has been developed. The ASD data reported in this article are based on the last follow-up visit. Therefore, high-quality studies with more extended follow-up periods are needed to prove this view.

This meta-analysis has several limitations. First, the sample sizes of part of the included articles were small. Studies with a small sample size are more likely to overestimate clinical effects. Second, although the same procedure was performed on patients in both the experimental and control groups, differences in the surgeon's experience, the extent of the patient's pathology, and scoring instruments in different studies still led to high heterogeneity in some of the indicators. Finally, the research was based on the statistics from published studies. The publication bias was unavoidable because of the limitations of a comprehensive review. The above conclusions may be not applicable to all patients.

## 5. Conclusion

This study shows that DCDA and CDA have similar results in terms of NDI scores, VAS scores, and EQ-5D scores. Also, they did not show significant differences in dysphagia. However, DCDA had a higher incidence of reoperation and fewer ASD than CDA. More high-quality studies are needed to provide more reliable data to compare the two treatments.
